# Association Between Ultraprocessed Food Consumption and Metabolic Disorders in Children and Adolescents with Obesity

**DOI:** 10.3390/nu16203524

**Published:** 2024-10-17

**Authors:** Gyeong-yoon Lee, Joo Hyun Lim, Hyojee Joung, Dankyu Yoon

**Affiliations:** 1 Division of Endocrine and Kidney Disease Research, Department of Chronic Disease Convergence Research, National Institute of Health, Cheongju 28159, Republic of Korea; gyounlee@korea.kr (G.-y.L.); mikorio@korea.kr (J.H.L.); 2 Department of Public Health, Graduate School of Public Health, Seoul National University, Seoul 08826, Republic of Korea; 3 Institute of Health and Environment, Seoul National University, Seoul 08826, Republic of Korea

**Keywords:** ultraprocessed food, metabolic disorders, MASLD, hepatic steatosis, insulin resistance, pediatric nutrition

## Abstract

Background/Objectives: We investigated the effects of ultraprocessed food (UPF) consumption on metabolic disorders (e.g., adiposity, metabolic associated steatotic liver disease [MASLD], and insulin resistance) in children and adolescents with obesity to improve dietary guidelines and public health strategies. Methods: The dietary intake of 149 participants (aged 8–17 years) was assessed with food diaries. The NOVA classification system was used to classify food according to the degree of processing. Metabolic outcomes, including the fat mass index (FMI), hepatic fat percentage, and insulin resistance, were measured via dual-energy X-ray absorptiometry (DXA), magnetic resonance imaging proton density fat fraction (MRI-PDFF), and biochemical analysis, respectively. Results: Greater UPF consumption from baseline to the 6-month follow-up was significantly associated with increased insulin and decreased total cholesterol and LDL-cholesterol. UPF consumption was positively associated with the prevalence of MASLD (liver MRI-PDFF ≥ 5%; odds ratio _T3 vs. T1_ = 1.75; 95% confidence interval [CI] 1.03, 3.00), moderate-to-severe MASLD (liver MRI-PDFF ≥ 10%; OR _T3 vs. T1_ = 4.19; 95% CI 1.72, 10.22), and insulin resistance (OR _T3 vs. T1_ = 2.44; 95% CI 1.33, 4.48), after adjusting for covariates. A linear dose-response relationship was observed between UPF consumption and the odds of moderate-to-severe MASLD and insulin resistance. Conclusions: Greater UPF consumption was strongly associated with MASLD and insulin resistance in children and adolescents with obesity, underscoring the importance of reducing UPF consumption through dietary guidelines and public health interventions to mitigate the risk of obesity-related metabolic conditions in young populations.

## 1. Introduction

Ultraprocessed foods (UPFs) have become a predominant component in diets world-wide. These foods, which contain ingredients made from extracted or synthesized food substances such as oils, sugars, hydrogenated fats, and modified starches, are designed for convenience, with extended shelf lives, high palatability, and little preparation. Typically, UPFs are high in sugars, fats, and salts but low in essential nutrients such as fiber and vitamins, and their consumption is associated with various adverse health outcomes due to accompanying nutritional deficiencies and a tendency toward overconsumption [1,2,3,4,5]. UPFs can also trigger inflammatory responses across multiple metabolic pathways, potentially leading to insulin resistance and MASLD [6,7,8]. These complex interactions can dynamically shift based on changes in specific nutrient intake.

In recent years, dietary habits among Asians have shifted toward increased UPF consumption, mirroring trends observed in other regions [9]. From 2006 to 2019, ultraprocessed food and beverage sales per capita in South and Southeast Asia increased from 4 to 9 kg, from 7 to 17 L, respectively. This is concerning because of its potential link to various health risks. In regions with established UPF consumption patterns, such as Western countries, these foods dominate the food supply and are strongly correlated with increases in the prevalence of obesity, cardiovascular diseases, and metabolic disorders [3,10,11]. While the health risk of UPF consumption on metabolic health has been identified among adults in this region [12,13,14,15,16,17,18,19,20], it is not well-documented among children and adolescents in Asia.

The increasing prevalence of childhood and adolescent obesity is a significant public health challenge, as it is closely associated with the early onset of comorbidities traditionally observed in adults [21,22,23]. One such condition, metabolic associated steatotic liver disease (MASLD), is characterized by excessive fat accumulation in liver cells associated with metabolic risk factors, including obesity, type 2 diabetes, dyslipidemia, and hypertension, and can progress to more severe liver conditions, such as nonalcoholic steatohepatitis (NASH), fibrosis, and cirrhosis [23,24,25,26]. The term MASLD was redefined from nonalcoholic fatty liver disease (NAFLD) [27]. The global prevalence of MASLD in adolescents has been steadily increasing and as MASLD often occurs in obese children, understanding the dietary factors contributing to its development is crucial [25,26]. It is widely accepted that there are very complex interactions between the liver and other endocrine axes, including MASLD, obesity, and insulin resistance [28]. Obesity is a contributing factor to insulin resistance, which in turn leads to the accumulation of liver fat and further exacerbates insulin resistance. Insulin resistance also promotes the progression of MASLD [29].

The relationship between UPF consumption and metabolic disorders in adults is well documented; however, there is still a lack of research on this relationship in children and adolescents, especially in the Asian population. To our knowledge, very few studies have evaluated the relationship between UPF consumption and MASLD in children and adolescents. This study aims to fill this gap by providing specific data on the effects of UPF consumption in obese Korean children and adolescents via magnetic resonance imaging proton density fat fraction (MRI-PDFF).

## 2. Materials and Methods

This was a prospective study using data from the Intervention for Childhood and Adolescents Obesity via Activity and Nutrition (ICANN), an intervention study conducted at a university hospital in Korea between August 2019 and December 2020. The eligibility criteria included an age- and sex-specific body mass index (BMI) at or above the 85th percentile according to the 2017 Korean National Growth Charts. Exclusion criteria included the use of medications that affect weight, such as insulin or steroids. The original study sample included 234 children and adolescents aged 8–17 years (75 females and 159 males).

We used baseline and 6-month follow-up data from 160 participants who did not drop out of follow-up in this analysis. Among those, 3 participants were excluded because of missing or incomplete food diary information, and 8 participants had missing data on variables of interest. Ultimately, 149 participants with complete baseline and follow-up anthropometric measurements, dietary records, and questionnaires were included in this study.

### 2.1. UPF Consumption

At baseline and at follow-up, a 3-day food diary was sent to parents or caregivers. They were instructed to record all the food and beverage items the children consumed on those days, excluding water. The dietary data were based on a 2-day food diary for 49 children (32.9%), a 3-day diary for 100 children (67.1%) at baseline, a 2-day food diary for 44 children (29.5%), a 3-day diary for 104 children (69.8%), and a 5-day diary for 1 child (0.7%) at follow-up. Nutrient intakes were coded via the CAN-PRO 5.0 program (the Korean Society of Nutrition, Seoul, Republic of Korea). The UPFs consumed were classified in accordance with the NOVA food classification system, which groups foods and beverages depending on the extent and purpose of industrial processing and artificial additives [2,30]. Almost all the food and beverage items were assigned to one of the 4 NOVA categories: (1) unprocessed or minimally processed foods (UMPs); (2) processed culinary ingredients; (3) processed foods; and (4) ultraprocessed foods. Home-made and artisanal foods were identified and decomposed into their ingredients via standardized recipes. In cases of mixed foods where the recipe was uncertain, those foods were classified into group 3 as cooked foods [31]. In Korea, almost all cheese and bread are processed, so they were assigned to group (4).

The daily consumption of UPFs was described in gram percentages and energy percentages. The proportion of UPFs in the total dietary intake (% of grams per day) was considered the main exposure to avoid missing out on food that does not provide any energy, such as artificially sweetened beverages and other non-nutritional additives.

### 2.2. Anthropometric Measurements

Anthropometric measurements, including fat mass index (FMI; fat mass/height^2^, kg/m^2^), total body fat mass, trunk fat mass, percentage of total body fat (total body fat mass/total mass, %), percentage of trunk fat (trunk fat mass/total mass, %), lean mass, and percentage of hepatic fat, were conducted in the hospital [32]. Total body fat mass and trunk fat mass were measured via whole-body DXA (Lunar Prodigy Advance with pediatric software version encore 14.0, GE, Healthcare, Madison, WI, USA). Body weight was measured via a bioelectrical impedance analysis (BIA; Inbody 720 Body Composition Analyzer, BioSpace Co., Ltd., Seoul, Republic of Korea). BMI was computed as body weight (kg) divided by height squared (m^2^) [33]. 

### 2.3. Biochemical Assessment

Venous blood samples were collected by a nurse after at least ten hours of fasting. High-density lipoprotein cholesterol (HDL-C; mg/dL) and low-density lipoprotein cholesterol (LDL-C; mg/dL) were measured through homogeneous enzymatic colorimetric tests (Cobas 8000 c702, Roche, Basel, Switzerland). The triglyceride (TG; mg/dL), aspartate aminotransferase (AST; U/L), alanine aminotransferase (ALT; U/L), gamma-glutamyl transferase (γ-GT; U/L), total cholesterol (TC; mg/dL), and fasting plasma glucose (mg/dL) levels were determined via enzymatic assays (Cobas 8000 c702, Roche). Fasting plasma insulin (µU/mL) was measured via electrochemiluminescence immunoassay (Cobas 8000 e802; Roche). The homeostasis model assessment for insulin resistance (HOMA-IR) score was calculated as fasting insulin (µU/mL) × fasting glucose (mg/dL)/405. The HOMA-IR cutoff values used for insulin resistance were 2.67 for boys and 2.22 for girls in the prepubertal period and 5.22 for boys and 3.82 for girls in the pubertal period [34].

### 2.4. Hepatic Steatosis Data

The participants were imaged via a 3 Tesla MRI scanner (MAGNETOM Skyra; Siemens Healthcare, Erlangen, Germany). Hepatic fat (%) was measured by MRI-PDFF, which is an index of the degree of fat deposition in the liver parenchyma [35]. MASLD is defined as the presence of hepatic steatosis (liver fat fraction ≥ 5%) in conjunction with one cardiometabolic risk factor (CMRF) and no other discernible cause (24). Since obesity is one of the CMRFs and there were no drinkers, all participants with hepatic steatosis were classified as having MASLD. The prevalence of moderate-to-severe MASLD (yes/no) was defined by the criterion of a fat fraction ≥10% [36].

### 2.5. Other Variables

Parents, caregivers, and children were provided with a set of questionnaires to complete. Primary caregivers reported information on maternal education attainment, birth weight, daily screen time (TV, computer, smartphone, and tablets), and physical activity. Physical activity level was categorized via the Global Physical Activity Questionnaire (GPAQ) into low, moderate, and high levels [37]. The Tanner stage was determined by a family physician’s examination to assess puberty at baseline and follow-up.

### 2.6. Statistical Analysis

Medians with interquartile ranges (IQRs) for continuous variables and numbers with percentages (%) for categorical variables were reported for descriptive analysis of participants’ demographic, behavioral, and dietary characteristics. Most continuous variables do not have a Gaussian distribution. UPF consumption was categorized into sex-specific tertiles to account for differences in food consumption between sexes. Differences in characteristics by sex-specific tertiles of UPFs were compared via a one-way ANOVA, the Kruskal–Wallis test, or the χ^2^ test, as appropriate.

Generalized estimating equations (GEEs), an extension of generalized linear models, were used for the analysis of repeated measures to account for possible changes in UPF consumption and metabolic outcomes between baseline and follow-up [38,39].

We assessed the associations between UPF consumption and the mean difference in metabolic outcomes, as well as the odds of MASLD prevalence and insulin resistance prevalence.

Continuous variables with a nonnormal distribution were log-transformed for analysis. Changes in effect estimates were calculated when each variable was added to the model sequentially in a stepwise manner.

For these analyses, UPF consumption was used both as a categorical variable, with the first tertile (T1, lowest intake) as the reference group, and as a continuous variable (per 10% increase). The model was adjusted for sex, age, ‘time’, birth weight, maternal education attainment, screen time, physical activity level, and daily energy intake. Adiposity outcomes, metabolic parameters, and fatty liver-related factors were compared across tertiles of UPF consumption using a linear regression analysis with GEEs, and the adjusted regression coefficients and 95% confidence intervals (CIs) were calculated. Logistic regression analysis with the GEE was conducted to identify the association between UPF consumption and MASLD in 142 participants with complete hepatic fat data, and between UPF consumption and insulin resistance in 149 participants with complete blood samples. The odds ratios (ORs) and 95% CIs for the prevalence of MASLD and insulin resistance were calculated after adjusting for covariates.

Missing data of less than 5% for activity minutes per day or the number of activity days in a week were imputed to the median. The data were analyzed via SAS 9.4, and *p* < 0.05 was considered to indicate statistical significance.

## 3. Results

### 3.1. General Characteristics

A total of 149 participants (43 girls [28.9%] and 106 boys [71.1%]) were included in this analysis (Table 1), with a median of 146.5 months [IQR, 133–162]. The median birth weight was 3.4 kg, which was similar across the tertiles. Hepatic fat significantly differed among the different UPF consumption tertile groups (*p* = 0.023). The participants with greater UPF consumption were more prone to having a higher age in months, lower maternal education attainment, and longer screen time and were less likely to have high activity levels.

### 3.2. Diet and UPF Consumption

The daily food consumption according to the food processing level is presented in Table 2. UPFs provided 20.4% of the daily food intake and 25.6% of the daily energy intake. The contribution of UPFs to daily energy intake was 11.1% in T1 and 44.8% in T3, whereas UMPs contributed 71.9% in T1 and 44.1% in T3.

The subjects in T3 were more likely to have a higher total energy intake (kcal) and a lower fiber intake (g) than the other subjects were (Table 3). The subjects in T3 obtained greater percentages of energy from total fat and lower percentages of energy from carbohydrates, saturated fatty acids (SFAs), polyunsaturated fatty acids (PUFAs), and monounsaturated fatty acids (MUFAs). No difference between groups was found for other variables, including the contribution to energy from proteins (%) or sodium intake (g).

### 3.3. UPF Consumption and Metabolic Risk Disorders

The associations of UPF consumption tertiles with adiposity outcomes, metabolic parameters, and fatty-liver-related factors are presented in Table 4. Compared with T1, T3 had significantly lower TC (β = −0.034; 95% CI [−0.065, −0.003]), and LDL-C (β = −0.056; 95% CI [−0.103, −0.010]) levels and higher insulin (β = 0.133; 95% CI [0.005, 0.262]). No associations were shown for the other outcomes.

**Figure 1:**
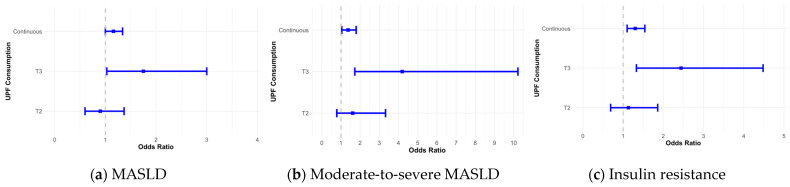
Associations between UPF consumption, MASLD, and insulin resistance in overweight children and adolescents (*n* = 149). The model was adjusted for month age, sex, ‘time’, maternal education attainment, birth weight, screen time, activity level, and daily energy intake. The continuous analysis was scaled for a 10% increase for easier interpretation. The HOMA-IR cutoff values for insulin resistance in the prepubertal period were 2.67 for boys, 2.22 for girls, and in the pubertal period, they were 5.22 for boys and 3.82 for girls. (**a**) Odds ratio of MASLD (hepatic fat ≥ 5%); (**b**) odds ratio of moderate-to severe MASLD (hepatic fat ≥ 10%); (**c**) odds ratio of insulin resistance.

Geater UPF consumption was positively associated with the prevalence of MASLD (OR _T3 vs. T1_ = 1.75; 95% CI [1.03, 3.00]), moderate-to-severe MASLD (OR _T3 vs. T1_ = 4.19; 95% CI [1.72, 10.22]), and insulin resistance (OR _T3 vs. T1_ = 2.44; 95% CI [1.33, 4.48]), after adjusting for total energy intake (Figure 1). We observed a direct linear dose–response relationship between a 10% increase in the proportion of UPFs consumed and the prevalence of moderate-to-severe MASLD (OR _continuous_ = 1.37; 95% CI [1.04, 1.79]) and insulin resistance (OR _continuous_ = 1.30; 95% CI [1.10, 1.54]), after adjusting for energy intake.

## 4. Discussion

This study aimed to explore the effects of UPF consumption on metabolic and liver health in obese children and adolescents. In this study, we present that greater UPF consumption was associated with elevated insulin levels and a greater prevalence of both insulin resistance and MASLD, particularly in its more severe forms, and significant reductions in TC and LDL-C levels, whereas other outcomes showed no significant associations.

We found that greater UPF consumption was significantly associated with elevated insulin levels and an increased prevalence of insulin resistance. The refined carbohydrates and high-glycemic (GI) index foods commonly found in UPFs can significantly raise insulin levels [4,40]. These foods rapidly elevate blood glucose, causing the body to release large amounts of insulin to return it back. Over time, frequent insulin spikes, particularly from refined sugars and rapidly digestible carbohydrates such as sugary products and white bread, can lead to insulin resistance [41].

One of the significant findings of this analysis was that greater UPF consumption was associated not only with the odds of MASLD but also with the odds of moderate-to-severe MASLD. This result indicates that UPF consumption significantly worsens the progression of hepatic steatosis and may contribute to more advanced stages of the disease. The disproportionate increase in the odds of severe MASLD suggests the possibility that certain characteristics of UPFs may directly worsen liver health, independent of overall caloric intake. Our findings align with those of previous cross-sectional studies. A recent study revealed that greater UPF consumption was positively associated with the prevalence of NAFLD in both adolescents (OR _Q5 vs. Q1_ = 2.34; 95% CI [1.01, 5.41]) and adults (OR _Q5 vs. Q1_ = 1.72; 95% CI [1.01, 2.93]) [42]. Several studies among adults have shown that greater UPF consumption is associated with increased odds of insulin resistance and NAFLD [43,44].

MASLD is a multisystem disease affecting various metabolic pathways and is strongly linked to insulin resistance and obesity [45,46]. Mechanistically, fat overload in the liver may lead to the accumulation of diacylglycerol, which activates protein kinase Cε (PKCε), impairing insulin signaling and leading to insulin resistance [22,47,48]. Body fat, particularly trunk fat, promotes insulin resistance through increased hepatic lipogenesis, leading to metabolic disturbances in the liver. When insulin resistance occurs, the oxidation of fatty acids in the liver decreases, promoting further fat accumulation and worsening hepatic steatosis. This process disrupts insulin signaling in the liver and increases fat accumulation, triggering inflammatory responses [49].

The negative effects of UPF consumption on metabolic disorders, such as the development of hepatic steatosis and insulin resistance, in children and adolescents can be explained through several mechanisms. First, the nutritional characteristics of UPFs may partially explain these effects. UPFs are energy-dense and contain added sugars (particularly fructose and sucrose), refined carbohydrates, and saturated and trans fatty acids while being low in fiber and vitamins, all of which are major risk factors for the development of metabolic disorders [50,51,52,53,54,55,56,57,58,59]. UPFs, which are rich in refined carbohydrates and sugars, can influence insulin levels and increase nutrient storage in adipose tissue [60]. Additionally, the low fiber content of UPFs impairs satiety signaling, leading to overconsumption [4]. Moreover, UPF consumption exposes individuals to potentially harmful substances added or created during industrial processing, such as sulfites, stabilizers, artificial additives, phthalates, and bisphenol A. These substances might contribute to the development of hepatic steatosis and other metabolic disorders [61,62,63,64,65]. Endocrine disruptors are believed to play a role in the pathogenesis of obesity and NAFLD [66]. Consequently, a diet high in UPFs often lacks diversity, replacing healthier options such as fruits, vegetables, whole grains, nuts, seeds, and a variety of unprocessed meats [67,68]. This imbalance can increase the risk of metabolic disorders [69,70] and promote conditions like adiposity and the prevalence of NAFLD [71,72]. The lack of fiber and fatty acids coupled with high UPF intake and high-GI food underscores how poor dietary diversity mediates the adverse metabolic outcomes associated with UPF consumption. 

In obese Korean children and adolescents, greater UPF consumption was associated with decreased levels of LDL-C and TC, as well as a lower contribution to energy from SFAs, PUFAs, MUFAs, and carbohydrates, despite greater overall energy intake. A notable characteristic of this population was that ready-to-eat/heat products, including processed meat, fish, and fried chicken, were the main UPFs consumed in the highest tertile of UPF consumption, followed by beverages, whereas unprocessed meat intake was lower than that in the other tertiles (data available upon request). Considering that higher UPF consumption was associated with increased energy intake, which is typically linked to elevated lipid levels, these findings were unexpected. However, it is possible that this reduction in LDL-C may be linked to the lower intake of SFAs, as saturated fat is known to increase LDL-C levels by inhibiting LDL receptor activity [73,74,75]. Additionally, the high intake of refined carbohydrates and high-GI foods may have contributed to this outcome, as previous studies have shown that substituting fats with high-GI carbohydrates can lower LDL-C and TC levels [76]. To further understand these patterns, comparisons with other populations offer valuable insights. A previous study in Ecuadorian adolescents presented that a ‘wheat-dense, animal-fat pattern’ was associated with higher blood cholesterol and LDL levels among rural participants (*p* = 0.02 and *p* = 0.04, respectively), whereas a ‘rice-rich, nonanimal fat pattern’ was linked to moderate increases in blood glucose levels among urban adolescents (*p* < 0.01) [77]. Another cross-sectional study of Iranian adults showed that individuals in the highest tertile of UPF consumption had significantly greater intakes of energy, carbohydrates, fats, and saturated fats, along with a greater consumption of processed and red meat, and breads (*p* < 0.01) [78]. In that study, greater UPF consumption was also associated with an increased odds ratio for HDL abnormalities, indicating a complex relationship between diet and lipid levels across different populations.

Building on these insights, excessive fat intake can exacerbate systemic inflammation and insulin resistance, leading to increased lipid accumulation in the liver, which can increase the risk of MASLD. This pathway is further supported by evidence that high fat consumption stimulates the release of pro-inflammatory cytokines like IL-6, TNF-α, and IL-1β, which triggers a systemic inflammatory response [6]. TNF-α impairs insulin action by activating JNK and IKKβ pathways, disrupting insulin signaling, while IL-1β and IL-6 exacerbate insulin resistance by inhibiting receptor functions and glucose uptake [7,8]. Consequently, this inflammatory cascade creates a feedback loop that worsens insulin resistance, impairing glucose regulation and increasing free fatty acids (FFAs) and hepatic fat accumulation, as documented in previous research [79]. High fat consumption has been found to promote lipid synthesis in the liver, worsening fatty liver conditions [80].

Importantly, UPFs are a major source of trans fatty acids, created through hydrogenation, forming partially hydrogenated oils (PHOs) used in industrial processing for their stability and cost-effectiveness [81]. Trans fats are linked to increased inflammation and impaired insulin signaling, raising the risk of metabolic diseases [82]. An animal study found trans fats significantly worsen metabolic conditions, like diabetes and fatty liver, more than saturated fats [83].

As mentioned earlier, metabolic pathways consist of multiple interacting physiological routes, making it challenging to predict metabolic outcomes using a single indicator. This complexity reinforces the importance of a comprehensive approach to assessing metabolic health, as single biomarkers may not adequately reflect the intricate interactions occurring in these physiological systems.

Despite the participants being obese, UPF consumption levels were lower than those of non-Asian children and adolescents, aligning with previous studies indicating a UPF consumption of 27.3% among the general young population in Japan [84]. Previous studies from Brazil, Spain, Portugal, and the UK reported that UPF consumption contributed approximately 30–50% of energy intake among children and adolescents [85,86,87,88,89]. Among Brazilian children, 34% had excess body weight, and 47% of their daily energy intake came from UPFs [90]. Our results showed that UPFs constituted a substantial portion of the daily energy intake among the highest UPF intake group, with levels comparable to those observed in Western regions. This broad distribution of UPF consumption suggests that, although overall levels are lower among Asian youth, those at the higher end of UPF intake face similar risks as Western youth do.

This study has several strengths, including its focus on a relatively underexplored population—Asian children and adolescents—and the use of MRI-PDFF, a highly accurate and reliable method for estimating liver fat percentage, which is considered the gold standard for hepatic fat quantification [91,92]. The use of food diaries instead of food frequency questionnaires (FFQs) also provides more detailed and reliable dietary intake data, capturing day-to-day variations in diet, reducing recall bias, and improving the accuracy of fluid intake estimates [93,94,95]. To our knowledge, this is the first study to evaluate and provide compelling evidence demonstrating the associations between UPF consumption and metabolic outcomes, including MASLD and insulin resistance, in obese youth. 

However, this study has several limitations, including its short duration, small sample size, and focus on a pediatric population with obesity, specifically in the Asian population, which may limit the generalizability of our findings. Additionally, the use of GEEs with data collected over a short interval may have constrained our ability to detect statistically significant associations, especially for slowly progressing markers such as obesity and liver fat. Future studies should consider longer-term observational periods and larger, more diverse sample sizes, with a greater number of participants, to confirm these findings and explore the long-term impacts of UPF consumption on metabolic health.

## 5. Conclusions

This study reveals a significant link between UPF consumption and the increased prevalence of MASLD and insulin resistance in Korean children and adolescents with obesity. Reducing UPF consumption could be a strategic goal to lower the prevalence and severity of MASLD and insulin resistance in pediatric populations. Moreover, subsequent research is needed to clarify the underlying mechanisms of these associations and to assess the effectiveness of interventions designed to reduce UPF consumption. In conclusion, comprehensive dietary guidelines and public health policies are essential to address the health challenges posed by UPF consumption among young populations.

## Figures and Tables

**Table 1:** Characteristics of participants across the tertiles of ultraprocessed food consumption (*n* = 149).

	Overall Population		Tertiles of Ultraprocessed Food Consumption ^1,2^						
Variables	(*n* = 298)		T1 (*n* = 98)		T2 (*n* = 99)		T3 (*n* = 101)		*p*-Value ^3^
Age, months, median (IQR)	146.5	(133.0, 162.0)	145	(133.0, 157.0)	145.0	(129.0, 160.0)	154.0	(135.0, 167.0)	0.072
Gender, N (%)									
Girl	86	(28.9)	28	(25.6)	30	(30.3)	28	(27.7)	0.919
Boy	212	(71.1)	70	(71.4)	69	(69.7)	73	(72.3)	
Screen time, N (%)									
<3 h	101	(33.9)	39	(39.8)	36	(36.4)	26	(25.7)	0.086
<4 h	70	(23.5)	25	(25.5)	24	(24.2)	21	(20.8)	
≥4 h	127	(42.6)	34	(34.7)	39	(39.4)	54	(53.5)	
Activity level, N (%)									
Low	106	(35.6)	32	(32.7)	41	(41.4)	33	(32.7)	0.045
Moderate	96	(32.2)	29	(29.6)	24	(24.2)	43	(42.6)	
High	96	(32.0)	37	(37.8)	34	(34.3)	25	(24.7)	
Birth weight, kg, median (IQR)	3.4	(3.1, 3.6)	3.4	(3.2, 3.6)	3.3	(3.0, 3.6)	3.4	(3.2, 3.7)	0.587
Maternal education, N (%)									
≤High school	60	(20.1)	18	(18.4)	17	(17.2)	25	(24.8)	0.274
University	182	(61.1)	56	(57.1)	64	(64.6)	62	(61.4)	
≥Graduate school	56	(18.8)	24	(24.0)	18	(18.2)	14	(13.9)	
Hepatic fat, %, median (IQR) ^4^	12.0	(5.5, 20.6)	8.4	(5.1, 19.5)	12.5	(5.5, 20.5)	13.9	(6.4, 22.0)	0.023
Total cholesterol, mg/dL, median (IQR) ^5^	164.0	(146.0, 183.0)	167.5	(147.0, 187.0)	164.0	(146.0, 184.0)	162.0	(145.0, 179.0)	0.549
Triglyceride, g/dL, median (IQR) ^5^	105.0	(77.0, 148.0)	96.0	(72.0, 139.0)	106.0	(77.0, 148.0)	113.0	(79.0, 155.0)	0.220
HDL-C, mg/dL, median (IQR) ^5^	48.0	(42.0, 55.0)	50.0	(42.5, 55.0)	48.0	(44.0, 57.0)	48.0	(42.0, 54.0)	0.468
LDL-C, mg/dL, median (IQR) ^5^	100.0	(85.0, 118.0)	101.0	(83.0, 118.0)	102	(88.0, 119.0)	97.0	(84.0, 118.0)	0.424
Glucose, mg/dL, median (IQR) ^5^	91.0	(86.0, 96.0)	91.0	(85.0, 97.0)	91.0	(86.0, 96.0)	91.0	(88.0, 97.0)	0.772

^1^ Data are presented as medians (interquartile ranges; IQRs) or counts (%). ^2^ Sex-specific tertiles of the proportion of ultraprocessed food consumed in the total quantity of daily food consumed. The cutoff values were 17%, 31% for girls and 14%, 26% for boys, respectively. ^3^ *p*-values for comparisons were tested by a one-way ANOVA, Kruskal–Wallis test, or χ^2^ test, as appropriate. ^4^ Total *n* = 141, boy *n* = 101, girls *n* = 40. ^5^ Total *n* = 148, boy *n* = 105, girls *n* = 43.

**Table 2:** Daily food consumption according to food processing level (*n* = 149).

	Overall Population		Tertiles of Ultraprocessed Food Consumption ^1,2^						
Variables	(*n* = 298)		T1 (*n* = 98)		T2 (*n* = 99)		T3 (*n* = 101)		*p*-Value ^3^
**% Grams, %g/d**									
Unprocessed or minimally processed foods	63.3	(51.7, 72.2)	74.0	(69.5, 80.1)	64.6	(57.7, 68.8)	49.1	(41.4, 54.3)	<0.001
Processed culinary ingredients	2.5	(1.7, 3.2)	3.0	(2.2, 3.9)	2.6	(1.9, 3.2)	1.8	(1.2, 2.7)	<0.001
Processed foods	10.9	(7.0, 16.1)	12.9	(8.5, 18.2)	12.4	(8.5, 16.3)	8.3	(4.6, 13.6)	<0.001
Ultraprocessed foods	20.4	(12.4, 32.4)	8.4	(5.2, 12.4)	20.3	(17.8, 23.5)	38.0	(32.3, 46.8)	<0.001
Other source ^4^	79.6	(67.4, 87.5)	91.3	(87.6, 94.8)	79.7	(76.5, 82.2)	62.0	(53.2, 67.7)	<0.001
**% Calories, %kcal/d**									
Unprocessed or minimally processed foods	58.7	(47.1, 69.1)	71.9	(66.5, 77.8)	58.3	(51.8, 64.4)	44.1	(37.7, 53.7)	<0.001
Processed culinary ingredients	6.2	(4.2, 8.3)	8.0	(6.0, 10.2)	6.0	(4.6, 7.7)	4.6	(2.9, 6.7)	<0.001
Processed foods	5.9	(2.4, 10.9)	6.4	(3.2, 11.8)	7.7	(3.8, 13.0)	3.7	(1.6, 9.1)	<0.001
Ultraprocessed foods	25.6	(14.4, 39.8)	11.1	(7.2, 16.8)	25.9	(20.7, 33.3)	44.8	(36.0, 51.3)	<0.001
Other source ^4^	74.4	(60.2, 85.6)	88.9	(83.2, 92.8)	74.1	(66.7, 79.3)	55.2	(48.7, 64.0)	<0.001

^1^ Data are presented as medians (IQRs). ^2^ Sex-specific tertiles of the proportion of ultraprocessed food consumed in the total quantity of daily food consumed. The cutoff values were 17%, 31% for girls and 14%, 26% for boys, respectively. ^3^ *p*-values for comparison were tested by Kruskal–Wallis test. ^4^ The sum of the three other food processing levels.

**Table 3:** Dietary intake contributions of subjects across tertiles of ultraprocessed food consumption (*n* = 149).

	Overall Population		Tertiles of Ultraprocessed Food Consumption ^1,2^						
Variables	(*n* = 298)		T1 (*n* = 98)		T2 (*n* = 99)		T3 (*n* = 101)		*p*-Value ^3^
Total energy, kcal	1916.6	(1547.5, 2263.4)	1738.5	(1455.7, 2086.1)	1966.7	(1612.8, 2255.0)	2111.6	(1606.1, 2405.0)	0.002
Contributions to energy, %									
Carbohydrates	52.8	(47.8, 58.7)	55.9	(51.1, 59.8)	52.8	(45.2, 58.4)	51.3	(46.7, 56.4)	0.006
Proteins	15.4	(14.1, 17.6)	15.4	(14.2, 17.2)	15.4	(13.9, 17.5)	15.4	(14.1, 18.4)	0.801
Total fat	29.8	(25.2, 34.4)	27.4	(23.7, 31.4)	29.8	(25.5, 35.1)	31.7	(27.3, 35.3)	<0.001
SFAs	5.2	(3.6, 6.7)	5.9	(4.4, 7.9)	5.2	(3.5, 6.3)	4.6	(3.2, 6.0)	<0.001
MUFAs	6.3	(4.4, 8.0)	7.5	(5.6, 9.1)	5.6	(4.0, 7.6)	5.6	(4.0, 7.2)	<0.001
PUFAs	5.5	(3.9, 6.9)	6.2	(5.2, 8.0)	5.1	(3.6, 6.6)	4.6	(3.0, 6.1)	<0.001
Protein intake per body weight, g/kg	1.1	(0.8, 1.4)	1.0	(0.8, 1.3)	1.1	(0.8, 1.4)	1.1	(0.8, 1.3)	0.410
Fiber, g	16.2	(12.7, 20.6)	17.1	(13.9, 21.8)	16.6	(13.3, 20.2)	14.6	(10.7, 19.1)	0.006
Sodium, g	3.1	(2.4, 3.9)	3.0	(2.2, 3.8)	3.1	(2.5, 3.8)	3.4	(2.5, 4.2)	0.116

Abbreviations: Saturated fatty acids (SFAs), Monounsaturated fatty acids (MUFAs), Polyunsaturated fatty acids (PUFAs). ^1^ Data are presented as medians (IQRs). ^2^ Sex-specific tertiles of the proportion of ultraprocessed food consumed in the total quantity of daily food consumed. The cutoff values were 17%, 31% for girls and 14%, 26% for boys, respectively. ^3^ *p*-values for comparison were tested by a one-way ANOVA or Kruskal–Wallis test, as appropriate.

**Table 4:** Associations between tertiles of ultraprocessed food consumption and metabolic outcomes (*n* = 149).

	Tertiles of Ultraprocessed Food Consumption ^1^ β Coefficient (95% CI)						Continuous ^2^ β Coefficient (95% CI)	
Variables ^3,4^	T1 (*n* = 98)		T2 (*n* = 99)		T3 (*n* = 101)			
FMI, kg/m^2^	0	(reference)	0.006	(−0.022, 0.034)	0.004	(−0.027, 0.035)	0.003	(−0.005, 0.010)
Body fat mass, kg	0	(reference)	0.002	(−0.026, 0.030)	0.003	(−0.028, 0.035)	0.002	(−0.005, 0.010)
Body fat percentage, %	0	(reference)	0.033	(−0.584, 0.649)	−0.100	(−0.804, 0.605)	−0.014	(−0.186, 0.158)
Trunk fat mass, kg	0	(reference)	0.001	(−0.031, 0.032)	0.004	(−0.031, 0.038)	0.002	(−0.006, 0.010)
Trunk fat percentage, %	0	(reference)	0.246	(−0.527, 1.019)	−0.035	(−0.895, 0.825)	−0.001	(−0.210, 0.208)
Lean mass, kg	0	(reference)	0.003	(−0.010, 0.015)	0.009	(−0.005, 0.023)	0.004	(0.0003, 0.007)
Hepatic fat, % ^5^	0	(reference)	0.043	(−0.063, 0.148)	0.127	(−0.010, 0.263)	0.028	(−0.008, 0.063)
AST, U/L ^6^	0	(reference)	−0.005	(−0.081, 0.072)	0.043	(−0.052, 0.139)	0.007	(−0.020, 0.035)
ALT, U/L ^6^	0	(reference)	0.036	(−0.084, 0.156)	0.082	(−0.064, 0.228)	0.012	(−0.032, 0.056)
γ-GTP, U/L ^6^	0	(reference)	0.026	(−0.037, 0.090)	0.068	(−0.008, 0.145)	0.014	(−0.010, 0.038)
Total cholesterol, mg/dL ^6^	0	(reference)	−0.013	(−0.046, 0.020)	−0.034	(−0.065, −0.003)	−0.010	(−0.018, −0.002)
Triglyceride, g/dL ^6^	0	(reference)	0.011	(−0.088, 0.109)	0.055	(−0.048, 0.159)	0.008	(−0.021, 0.036)
HDL cholesterol, mg/dL ^6^	0	(reference)	−0.001	(−0.035, 0.033)	−0.010	(−0.046, 0.026)	−0.003	(−0.012, 0.007)
LDL cholesterol, mg/dL ^6^	0	(reference)	−0.015	(−0.060, 0.030)	−0.056	(−0.103, −0.010)	−0.016	(−0.028, −0.004)
Glucose, mg/dL ^6^	0	(reference)	−0.013	(−0.039, 0.012)	0.01	(−0.02, 0.04)	−0.0004	(−0.01, 0.01)
Insulin, μU/mL ^6^	0	(reference)	−0.001	(−0.122, 0.121)	0.133	(0.005, 0.262)	0.032	(−0.001, 0.065)
HOMA-IR ^6,7^	0	(reference)	−0.020	(−0.155, 0.115)	0.133	(−0.005, 0.271)	0.029	(−0.008, 0.066)

Abbreviations: Fat mass index (FMI), metabolic-associated steatotic liver disease (MASLD), confidence intervals (CIs). ^1^ Sex-specific tertiles of the proportion of ultraprocessed food consumed in the total quantity of daily food consumed. The cutoff values were 17%, 31% for girls and 14%, 26% for boys, respectively. ^2^ The scale was 10%/day increase for better interpretation. ^3^ Data were log-transformed, except body fat percentage and trunk fat percentage. ^4^ Adjusted for month age, sex, ‘time’, maternal education attainment, birth weight, screen time, activity level, and daily energy intake. ^5^ Total *n* = 141, boy *n* = 101, girls *n* = 40. ^6^ Total *n* = 148, boy *n* = 105, girls *n* = 43. ^7^ The HOMA-IR cutoff values for insulin resistance in the prepubertal period were 2.67 for boys, 2.22 for girls, and in the pubertal period, they were 5.22 for boys, 3.82 for girls.

## Data Availability

The data presented in this study are available upon request from the corresponding author. The data are not publicly available due to privacy or ethical restrictions.
